# The role of cerclage wiring in the management of subtrochanteric and reverse oblique intertrochanteric fractures: a meta-analysis of comparative studies

**DOI:** 10.1007/s00590-022-03240-z

**Published:** 2022-03-21

**Authors:** Ashraf T. Hantouly, Motasem Salameh, Ahmad A. Toubasi, Loay A. Salman, Osama Alzobi, Abdulaziz F. Ahmed, Ghalib Ahmed

**Affiliations:** 1 Department of Orthopaedic Surgery, Surgical Specialty Center, Hamad Medical Corporation, Doha, Qatar; 2 Orthopedic Surgery Department, Warren Alpert Medical School of Brown University, Providence, RI USA; 3 Faculty of Medicine, The University of Jordan, Amman, Jordan; 4 Department of Orthopaedic Surgery, Johns Hopkins School of Medicine, Baltimore, MD USA

**Keywords:** Subtrochanteric fractures, Intertrochanteric, Reverse oblique fractures, Femur, Intramedullary nail, Cerclage

## Abstract

**Purpose:**

Subtrochanteric and reverse oblique intertrochanteric fractures are challenging and often difficult to reduce. While intramedullary nailing (IMN) is considered the standard treatment, achieving anatomic reduction prior to fixation is essential. This study aimed to assess the impact of cerclage wiring with IMN on the outcomes and complication rate in treating subtrochanteric and reverse oblique intertrochanteric fractures.

**Methods:**

This meta-analysis was conducted in line with PRISMA guidelines. The primary outcome was the time to union. The secondary outcomes were operative time, blood loss, quality of reduction, reduction alignment (if in varus), complications and reoperations. PubMed, Cochrane, Web of Science and Google Scholar were searched till July 2021. Articles that compared intramedullary nailing (IMN) versus intramedullary nailing and cerclage wiring (IMN-C) in the treatment of subtrochanteric and reverse oblique intertrochanteric fractures were included. The risk of bias was assessed using the Newcastle–Ottawa scale.

**Results:**

This meta-analysis included 415 patients with subtrochanteric and reverse oblique intertrochanteric fracture from six comparative studies. Our findings showed that IMN-C was significantly associated with higher mean duration of surgery and blood loss. However, IMN-C had significantly lower mean time to union compared to IMN alone. In addition, IMN-C had lower pooled prevalence of varus reduction and overall complications.

**Conclusion:**

This study showed that the use of cerclage wiring is associated with lower time to union, lower prevalence of varus reduction and overall complications. Therefore, cerclage wiring augmentation is a safe technique with low complication rate and may be advised whenever open reduction is needed in the management of subtrochanteric and reverse oblique intertrochanteric fractures.

**Supplementary Information:**

The online version contains supplementary material available at 10.1007/s00590-022-03240-z.

## Introduction

Hip fractures are one of the leading causes of morbidity and loss of disability-adjusted life years (DALYs) worldwide, with an enormous economic burden [12, 21]. In the USA alone, 300,000 patients are hospitalized each year due to hip fractures, resulting in more than 17 billion dollars bills in treatment [7]. Owing to the progressively aging populations, particularly in Western nations, these numbers are projected to continue to increase to reach 6.26 million annual cases worldwide by 2050 [6, 13].

Subtrochanteric fractures contributed to about 7–34% of all femur fractures [3]. These injuries are often associated with high-energy trauma (MVC) in young patients and low energy (e.g., falls) in the elderly [20]. Biomechanically, these fractures are quite challenging in terms of stability due to the interplay of internal (powerful hip muscle contractions) and external (Body weight and gravity) acting forces. Similarly, reverse obliquity intertrochanteric fracture patterns are common unstable patterns that pose a mechanical challenge [8]. Due to advanced designs, intramedullary nailing (IMN) is now the mainstay treatment for fixing most subtrochanteric and reverse oblique intertrochanteric fractures. Anatomic reduction before fixation is key in these unstable patterns; however, due to the high degree of instability, achieving and maintaining a good reduction alignment are not always feasible, resulting in poor outcomes with nonunion, malunion and implant failure [9, 17]. Furthermore, several studies have supported using cerclage wiring along with IMN to aid in the anatomic reduction of unstable peri-trochanteric fractures; however, low power with small sample sizes and short-term follow-up were some of the setbacks [16, 17]. Also, concerns of periosteal blood circulation and potential bone healing disruptions associated with the use of cerclage wiring have been described in the literature and remain controversial [2, 14, 27]. Therefore, high-quality evidence is needed to highlight the effect of cerclage wiring on clinical and radiological outcomes of surgical fixation of such fractures.

This meta-analysis aimed to study the impact of cerclage wiring with intramedullary on the outcomes and complication rate in the treatment of subtrochanteric and reverse intertrochanteric oblique fractures. We hypothesize that there is no significant difference in outcomes and complication rates between patients treated with cerclage wiring and IMN versus those treated with IMN alone.

## Materials and methods

We conducted this meta-analysis with adherence to the Preferred Reporting Items for Systematic Reviews and Meta-Analyses (PRISMA) guidelines [19]. The focus was studies that compared intramedullary nailing (IMN) alone and intramedullary nailing with cerclage (IMN-C) in the management of subtrochanteric and reverse oblique intertrochanteric fractures. The primary outcome was the time to union. The secondary outcomes included operative time, blood loss, quality of reduction, reduction alignment (if in varus), complications and reoperation rate.

## Eligibility criteria

Accessible articles published in English literature that compared intramedullary nailing with intramedullary nailing and cerclage wiring in the treatment of subtrochanteric and intertrochanteric reverse oblique fractures, as per OTA classification we included in this study [18].

## Exclusion criteria

Non-comparative studies, which reported only one of the two modalities of treatment, biomechanical and technical studies, were excluded. Studies that included pathological fractures, atypical fractures, hip fractures other than the intertrochanteric reverse oblique and subtrochanteric fractures and fractures treated with implants other than IMN and IMN-C were not included. We only included accessible articles that were published in English.

## Information sources and search strategy

PubMed, Cochrane, Web of Science and Google Scholar were searched till July 2021. The following keywords were used in the search: “Subtroch*” AND “Femur” AND “Fracture” AND “Nail” AND “Cerclage”. The studies were screened by titles and abstracts, and the full-text review was done once the study was eligible as per the above-mentioned criteria. Two authors performed the search strategy independently, and the senior author resolved any disagreement.

## Data collection process and data items

The collected data items include the following: author’s name, study year, country of origin, age, sex, sample size, fracture type, time to union, blood loss, operative time, quality of reduction, reduction alignment, follow-up duration, complications and reoperation rate. Two independent authors performed the data collection, with any disagreement being resolved by a senior author.

## Risk of bias in individual studies

The qualitative analysis was performed using the Newcastle–Ottawa scale (NOS) [24]. The tool contains three domains that are assess selection, comparability and outcome. Each study was assessed with the NOS by three authors independently. The final rating of each study was reviewed by the three authors and the senior author to reach a consensus.

## Statistical analysis

Meta XL, version 5.3 (EpiGear International, Queensland, Australia), was used for quantitative synthesis. Treatment effects were estimated by calculating the prevalence with 95% confidence intervals (CI) for dichotomous variables and the mean difference (MD) with 95% CI for continuous variables. For studies not reporting SD, we used the Cochrane Hand book for Systematic Reviews of Interventions for SD calculation from the 95% CI. For studies reporting medians and interquartile ranges instead of mean values and SD, we applied the conversion formula reported by Hozo et al. because we had no assumption of the data distribution [11]. Heterogeneity among studies was assumed to be present because of difference in study methods and outcomes definition. Studies were reweighted based on the inverse variance and pooled by a random-effect model. Cochran's Q heterogeneity test and *I*^2^ statistic were used to assess statistical heterogeneity.

## Results

### Study selection and patient characteristics

**Fig. 1:**
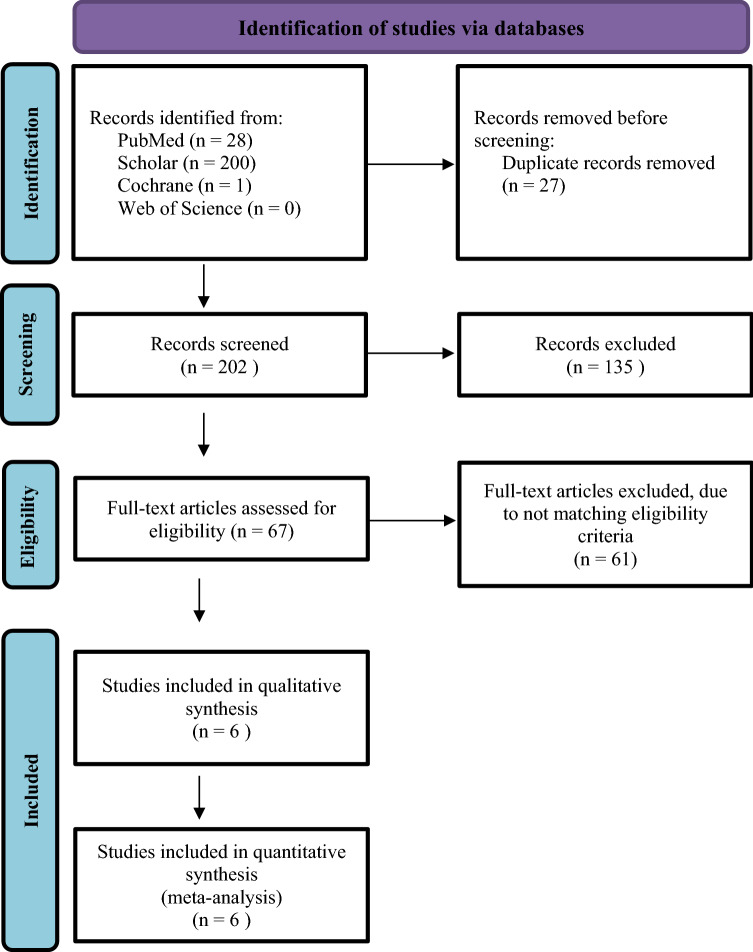
Search strategy flowchart

The search strategy yielded 229 articles, 27 of them were duplicates. The remaining 202 articles were screened using title and abstract, of which 135 were excluded. The lasting 67 articles were reviewed in full text. Subsequently, 61 were excluded and only six articles were eligible for inclusion in the meta-analysis. The PRISMA flowchart is displayed in Fig. 1. A total of 415 patients were included in this meta-analysis. IMN without cerclage was utilized in 71.8% of patients (*n* = 298), whereas 28.2% (*n* = 117) had IMN-C. The characteristics of the included studies are summarized in Table 1.

**Table 1:** Characteristics of included studies

Study	Country	Group	Age	Male (N)	Surgery duration	Blood loss (mL)	Time to union (weeks)	Reduction quality IMN-C	Reduction quality IMN	Open reduction without cerclage	Number of cerclage wires	Classification	Follow-up (months)
Annapa [1]	India	IMN-C 14 IMN 41	56.4	NR	NR	NR	NR	Good 9 Acceptable 3 Poor 2	Good 9 Acceptable 23 Poor 9	16	NR	NR	18
Patil [22]	India	IMN-C 15 IMN 19	48.9 53.5	9 12	96.8 78.8	180 120	14.5 15.6	Good 14 Acceptable 0 Poor 1	Good 15 Acceptable 2 Poor 2S	NR	1 or 2	NR	15.8
Bhat [4]	India	IMN-C 36 IMN 17	62.3	54 46	55.2 45	80 50	15.2 17.2	Good 14 Acceptable NR Poor NR	Good 21 Acceptable NR Poor NR	0	NR	31A3.1 Boyd Griffin type 3 Evan’s type 2	12
Tricka [22]	India	IMN-C 21 IMN 27	49.9 50.52	9 14	104.47 87.59	200 150	17.4 18.15	Good 20 Acceptable NR Poor NR	Good 20 Acceptable NR Poor NR	NR	1 or 2	32A3.1.1 32A2.1 32B1.1 32C1.1	20.8
Hoskins [10]	Australia	IMN-C 20 IMN 115	69 62	13 40	NR	NR	NR	Good 12 Acceptable NR Poor NR	Good 20 Acceptable NR Poor NR	45	NR	NR	4
Codesido [5]	Spain	IMN-C 30 IMN 60	81.97 84.3	8 9	100.69 84.53	NR	17.4 27.6	Good 29 Acceptable 1 Poor 0	Good 24 Acceptable 20 Poor 16	0	1 or 2	31A3 32A1 32A2 32B1 32B2 32C1	24

*NR* Not reported

### Quality assessment

The six prospective cohort studies scored three stars for the selection domain. Codesido et al., Trikha et al. and Patil et al. scored the maximum of two stars for the comparability domain [5, 22, 26]. Regarding the outcome domain, Codesido et al., Trikha et al. and Annappa et al. scored the maximum of three stars, Baht et al. and Patil et al. scored two stars, and Hoskins et al. scored one star [1, 4, 5, 10, 22, 26]. A summary of the qualitative assessment, according to the Newcastle–Ottawa scale, is shown in Supplementary Table1.

### Operative time and union time

**Fig. 2:**
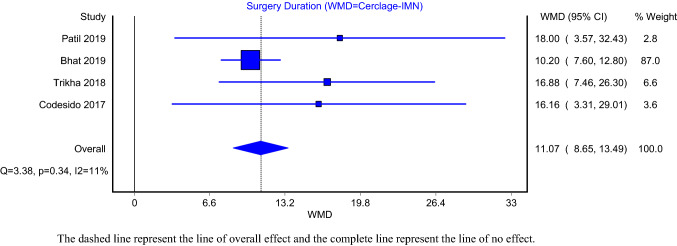
Surgery duration

**Fig. 3:**
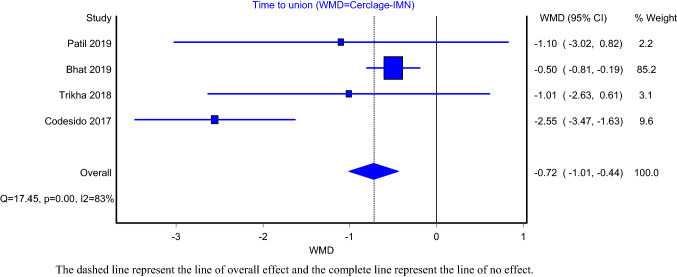
Time-to-union

The comparison models of operative time and union time included four articles. The analysis demonstrated that subtrochanteric fractures treated with IMN-C had significantly higher mean operative time compared to IMN alone (Fig. 2; WMD = 11.07; 95%CI: 8.65–13.49). The heterogeneity of this model was not significant (*I*^2^ = 11%; *P* value > 0.05). Intramedullary nailing of subtrochanteric fracture with cerclage wiring had significantly lower time to union (Fig. 3;WMD = −0.72; 95%CI:- − 1.01– − 0.44). The heterogeneity of this model was significant (*I*^2^ = 83%; *P* value < 0.05). Furthermore, the models assessed delayed union included five articles in the IMN-C and six articles in the IMN alone. The pooled prevalence of delayed union in the intramedullary nailing with and without cerclage wiring was 6% (Supplementary Fig. 1; 95%CI: 0–15%) and 10% (Supplementary Fig. 2; 95%CI: 6–16%), respectively. The heterogeneity of both the IMN-C (*I*^2^ = 51%; *P* value = 0.08) and IMN (*I*^2^ = 17%; *P* value = 0.31) models was insignificant.

The dashed line represents the line of overall effect, and the complete line represents the line of no effect.

The dashed line represents the line of overall effect, and the complete line represents the line of no effect.

### Fracture reduction status

**Fig. 4:**
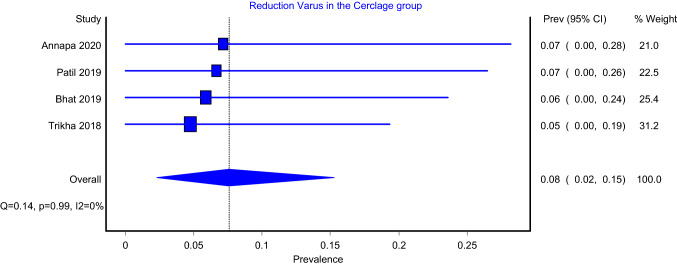
Varus reduction in IMN-C group

**Fig. 5:**
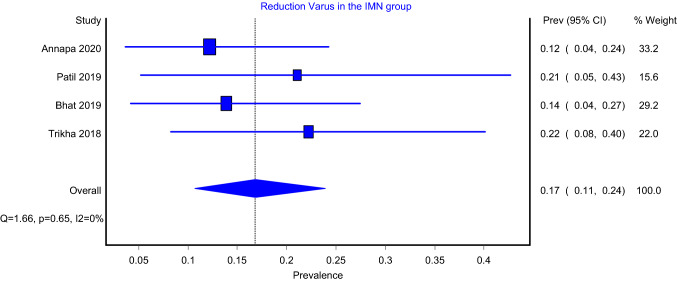
Varus reduction in IMN group

The prevalence models of reduction status, which included four articles, showed that the pooled prevalence for varus reduction for intramedullary nailing with and without the use of cerclage wiring was 8% (Fig. 4; 95%CI: 2–15%) and 17% (Fig. 5; 95%CI: 11–24%), respectively. The heterogeneity of both models was insignificant (*I*^2^ = 0%; *P* value > 0.05). Furthermore, the pooled prevalence model which included six articles showed that good reduction status for intramedullary nailing with and without cerclage wiring were 84% (Supplementary Fig. 3; 95%CI: 70–95%) and 47% (Supplementary Fig. 4; 95%CI: 26–86%), respectively. The heterogeneity of the IMN-C (*I*^2^ = 70%; *P* value < 0.05) and IMN (*I*^2^ = 92%; *P* value < 0.05) models were significant. In addition, the pooled prevalence for acceptable reduction for the intramedullary nailing with and without the use of cerclage wiring was 7% (Supplementary Fig. 5; 95%CI: 0–20%) and 33% (Supplementary Fig. 6; 95%CI: 12–85%), respectively. Both the IMN-C (*I*^2^ = 58%; *P* value < 0.05) and IMN (*I*^2^ = 85%; *P* value < 0.05) models showed significant heterogeneity. Moreover, the pooled prevalence for poor reduction for the IMN was 5% with significant heterogeneity (Supplementary Fig. 7; 95%CI: 0–14%; *I*^2^ = 39%; *P* value < 0.18), whereas it was 21% in the IMN group with insignificant heterogeneity (Supplementary Fig. 8; 95%CI: 15–28%; *I*^2^ = 0%; *P* value < 0.42).

### Blood loss

**Fig. 6:**
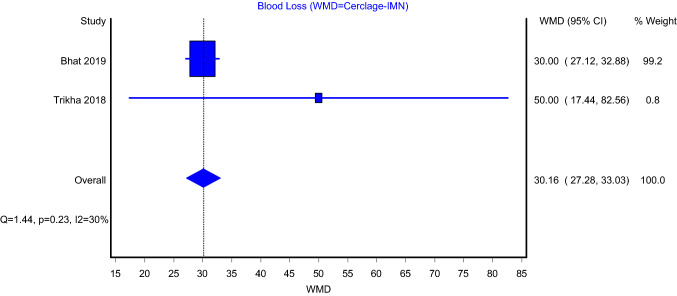
Blood loss

The mean blood loss comparison model between intramedullary nailing with and without cerclage wiring included two articles and showed that there is a higher mean blood loss with the use of cerclage (Fig. 6; WMD = 30.16; 95%CI: 27.28–33.03). This model showed insignificant heterogeneity (*I*^2^ = 30%; *P* value = 0.23).

### Complications

**Fig. 7:**
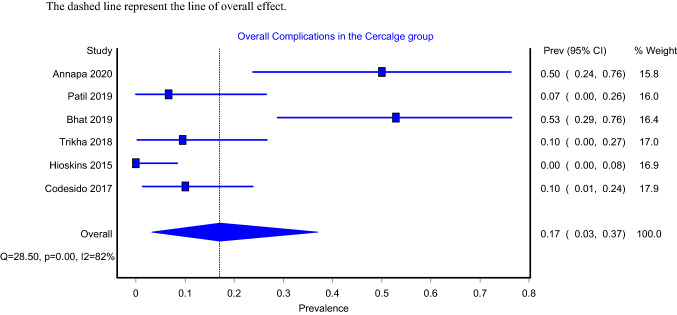
Overall complications in IMN-C group

**Fig. 8:**
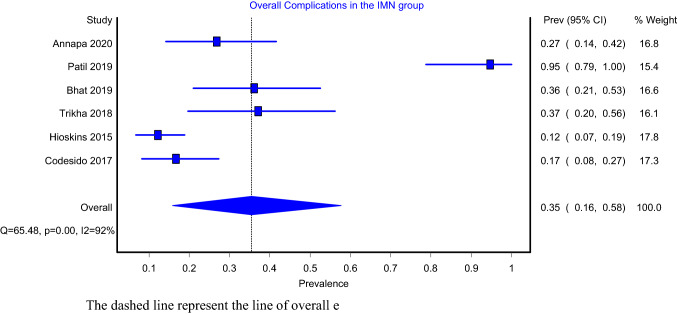
Overall complications in IMN group

Models that assessed for overall complication included six articles and showed that the pooled prevalence for intramedullary nailing with and without cerclage wiring were 17% (Fig. 7; 95%CI: 3–37%) and 35% (Fig. 8; 95%CI: 16–68%), respectively. Both the IMN-C (*I*^2^ = 82%; *P* value = 0.00) and the IMN alone (*I*^2^ = 92%; *P* value = 0.00) models showed significant heterogeneity. Complications reported by each study are shown in Table 2.

**Table 2:** Complications reported in the included studies

Study	Group	Superficial infection	Deep infection	Varus reduction	Leg length discrepancy	Implant failure	Screw cutout	Screw back-out	Loss of fixation	Delayed union/nonunion	Reoperations
Annapa [1]	IMN-C IMN	* 1	2 2	1 5	* *	2 3	* *	* *	* *	3 5	* *
Patil [22]	IMN-C IMN	1 0	0 1	1 4	0 2	0 3	0 3	0 5	* *	* 4	0 3
Bhat [4]	IMN-C IMN	4 2	4 1	1 5	* *	* *	1 3	0 5	* *	0 2	5 4
Tricka 2018	IMN-C IMN	0 0	0 4	1 6	1 4	* 2	* *	* *	* *	1 4	1 4
Hoskins [10]	IMN-C IMN	* *	* 0	* *	* *	0 2	0 2	* *	5 3	0 7	0 13
Codesido [5]	IMN-C IMN	2 2	0 0	* *	* *	* *	1 3	* *	* *	0 5	0 4

*Not mentioned or not clearly stated

1. Infection
  The superficial infection prevalence models included four articles. This model revealed that the prevalence of superficial infection in intramedullary nailing with cerclage wiring was 8% (Supplementary Fig. 9; 95%CI: 1–19%) and the heterogeneity of this model was insignificant (*I*^2^ = 55%; *P* value = 0.08). However, the superficial infection pooled prevalence in the intramedullary nailing without cerclage wiring was 3% (Supplementary Fig. 10; 95%CI: 1–6%) and the heterogeneity of this model was insignificant (*I*^2^ = 0%; *P* value = 0.69). In addition, the pooled deep infection prevalence was 6% in the intramedullary nailing with cerclage wiring (Supplementary Fig. 11; 95%CI: 0–16%) while it was 2% in the intramedullary nailing without cerclage wiring (Supplementary Fig. 12; 95%CI: 0–6%). The heterogeneity of the deep infection IMN-C model (*I*^2^ = 68%; *P* value = 0.00) was significant while it was insignificant for the IMN alone model (*I*^2^ = 22%; *P* value = 0.27).
2. Leg length discrepancy
  The leg length discrepancy model included two articles. The pooled prevalence of leg length discrepancy in the intramedullary nailing with and without cerclage wiring were 4% (Supplementary Fig. 13; 95%CI: 0–12%) and 14% (Supplementary Fig. 14; 95%CI: 5–25%), respectively. The heterogeneity of both models was insignificant (*I*^2^ = 0%; *P* value > 0.05).
3. Implant failure
  The implant failure models included four articles. The pooled prevalence of implant failure in the intramedullary nailing with cerclage wiring was 4% (Supplementary Fig. 15; 95%CI: 0–14%) and the heterogeneity of this model was insignificant (*I*^2^ = 47%; *P* value = 0.15). In the intramedullary nailing without cerclage wiring, the implant failure pooled prevalence was 6% (Supplementary Fig. 16; 95%CI: 1–14%) and the heterogeneity of this model was insignificant (*I*^2^ = 60%; *P* value = 0.06). The screw cutout prevalence model included four articles while the screw back-out model included two articles. Screw cutout and back-out pooled prevalence in the intramedullary nailing with cerclage wiring were 3% (Supplementary Fig. 17; 95%CI: 0–8%) and 1% (Supplementary Fig. 18; 95%CI: 0–7%), respectively, with the heterogeneity of both models was low (*I*^2^ = 0%; *P *value > 0.05). Nevertheless, screw cutout and back-out pooled prevalence in the intramedullary nailing without cerclage wiring were 6% with insignificant heterogeneity (Supplementary Fig. 19; 95%CI: 2–12%; *I*^2^ = 59%; *P* value = 0.06) and 19% with insignificant heterogeneity (Supplementary Fig. 20; 95%CI: 8–32%; *I*^2^ = 19%; *P* value = 0.27), respectively.
4. Reoperation and revision rate
  The models of reoperation and revision included five articles. The pooled prevalence of reoperation and revision for intramedullary nailing with cerclage wiring was 6% (Supplementary Fig. 21; 95%CI: 0–19%) with significant heterogeneity (*I*^2^ = 80%; *P* value = 0.00). The reoperation and revision pooled prevalence for intramedullary nailing without cerclage wiring was 14% (Supplementary Fig. 22; 95%CI: 10–18%) with insignificant heterogeneity (*I*^2^ = 0%; *P* value = 0.85).

## Discussion

In this meta-analysis on subtrochanteric fractures treated with IMN, cerclage wiring was associated with shorter time to union, lower rates of varus malreduction, lower incidence of implant failure and overall complications with lower need to reoperation. On the other hand, IMN without cerclage use was associated with shorter operative time, lower mean blood loss and decreased rates of superficial and deep infections.

Due to the characteristic anatomy and biomechanics, subtrochanteric fractures are considered a challenge to most orthopedic surgeons. High rates of varus malreduction were reported in the literature. Starr et al. in an RCT comparing piriformis versus trochanteric entry for the treatment of subtrochanteric fractures reported 17% over all varus malreduction and 38% good reduction with no difference between both entry portals [25]. This was comparable to this meta-analysis pooled prevalence of 17% of varus malreduction and 48% of good reduction using IMN without cerclage. Varus malreduction was reported to increase the risk of nonunion, malunion, implant failure and reoperation [15]. Anatomic reduction of subtrochanteric fractures was proved to improve the quality of life and functional outcomes of patients [23].

Percutaneous technique, clamp-assisted open reduction and open reduction and cerclage wiring are among the technique used by orthopedic surgeons to enhance the quality of reduction in subtrochanteric fractures. One can argue that the open reduction and clamping alone before nail insertion can be enough to ensure anatomic reduction, avoiding the risk of disrupting the periosteal blood circulation, the longer operative time and the higher blood loss using the cerclage wiring. In this review three articles (Trikha, Codesido and Patil) compared closed reduction with or without percutaneous techniques versus open reduction and cerclage wiring, two articles (Hoskins, Annappa) compared closed reduction or clamp-assisted open reduction versus open reduction and cerclage wiring [1, 5, 10, 22, 26]. And one article (Bhat et al.) included only cases with IMN after open reduction; Bhat et al., in the only prospective comparative study on the topic, compared open clamp-assisted reduction versus open reduction and cerclage wiring in reverse oblique intertrochanteric fractures [4]. They reported 14% varus malreduction and 6% nonunion rate in the no cerclage group compared to 6% varus malreduction and no nonunion reported in the cerclage group. In addition, they reported an anatomic reduction in 58% of the no cerclage and 82% of the cerclage group. Furthermore, the time to union was significantly shorter in the cerclage group (3.8 months vs 4.3 months) *P* = 0.0041, with a significantly higher Harris hip scores at final follow-up (*P* = 0.03).

Hoskins et al., in the largest cohort on the topic, included 135 cases of subtrochanteric fractures with 48.9% (66 cases) required open reduction, of which 20 patients (32.5%) were augmented with cerclage wiring [10]. The author reported no reoperation in the cerclage group compared to 15% in the open reduction and no cerclage group. The quality of reduction was significantly better in the cerclage group with lower fracture displacement and better angular deformity (*P* < 0.05).

Not all fracture configurations are amenable to cerclage wiring; this can be considered an important source of bias when comparing reduction techniques in subtrochanteric fractures. Three articles in this review included only fractures configuration that considered suitable for cerclage wiring (Bhat, Annappa, Trikha); Trikha et al. included long oblique, spiral or spiral wedge and comminuted fractures in their retrospective cohort, and quality of reduction was significantly better in the open reduction and cerclage group compared to closed reduction with shorter time to union and lower nonunion rate [1, 4, 26]. On the other hand, surgical time and blood loss were significantly higher in the cerclage group. Similarly, Annappa et al. in their retrospective cohort reported the need of open reduction in 54% of the cases (30/55 patients), of which 14 patients underwent cerclage wiring. Only fractures that were considered amenable to cerclage wiring were included [1]. The authors reported one case of varus malreduction in the cerclage group compared to 15 cases in the no cerclage group with no statistical significance. Moreover, cerclage wiring was associated with higher nonunion and infection rates that were statistically insignificant.

Limitations of this meta-analysis should be acknowledged. Like all other meta-analysis, there was heterogeneity among the included studies and the bias of the primary studies was unknown. We included articles that were published only in English, five of which were conducted retrospectively. Thus, selection bias could not be eliminated in such design and data collection was dependent on the accuracy of follow-up documentation. Another limitation is the small number of the included studies as our search strategy, which excluded non-comparative studies and those utilizing implants other than IMN and IMN-C, identified only six comparative studies in the literature to assess the desired outcome measures with a total of 415 patients. Furthermore, the low number of participants in the included articles limited our ability to conduct comparisons for the quality of reduction and complications using more reliable effect measures such as odds ratio as whenever we tried to do such analysis, we encountered very wide confidence intervals. Accordingly, since comparative analyses using odds ratios were not reliable, we used prevalence and its related confidence intervals. As a result, well-conducted prospective comparative studies with larger sample size are required for better assessment of the efficacy and safety of cerclage wiring. Quality of the included studies ranged between 5/9 and 8/9 as per the Newcastle–Ottawa Scale. Another important limitation is that subgroup analysis according to fracture type could not be done. Thus, future studies are recommended to report outcomes data for the fracture types. However, and to the best of our knowledge, this is the first meta-analysis to pool data from comparative studies on the topic. This information can be used for randomized control trials on the management of subtrochanteric and reverse oblique intertrochanteric fractures.

## Conclusion

This meta-analysis demonstrated that cerclage wiring augmentation with intramedullary nailing of subtrochanteric and reverse oblique intertrochanteric fractures is associated with lower time to union and lower prevalence of varus reduction and overall complication. Therefore, cerclage wiring is a safe technique with low complication rate and may be advised whenever open reduction is needed in the management of subtrochanteric and reverse oblique intertrochanteric fractures.

## Supplementary Information

Below is the link to the electronic supplementary material.

Supplementary file1 (DOCX 113 KB)

## References

[1] Annappa R, Kamath SU, Krishnamurthy SL, Mallya S, Kamath K, Suresh PK (2020). Does cerclage wiring with intramedullary nailing in subtrochanteric fractures improve the final outcome?. Medico Legal Update.

[2] Apivatthakakul T, Phaliphot J, Leuvitoonvechkit S (2013). Percutaneous cerclage wiring, foes it disrupt femoral blood supply? a cadaveric injection study. Int J Care Inj.

[3] Bedi A, Le Toan T (2004). Subtrochanteric femur fractures. Orthop Clin North Am.

[4] Bhat TA, Butt MF, Beigh IA, Mantoo SA, Ahmad MN, Ganie IA (2019). Comparative study of intramedullary nailing in reverse obliquity intertrochanteric fractures with or without cerclage wire augmentation. Int J Orthop Sci.

[5] Codesido P, Mejía A, Riego J, Ojeda-Thies C (2017). Subtrochanteric fractures in elderly people treated with intramedullary fixation: quality of life and complications following open reduction and cerclage wiring versus closed reduction. Arch Orthop Trauma Surg.

[6] Cooper C, Cole ZA, Holroyd CR, Earl SC, Harvey NC, Dennison EM (2011). Secular trends in the incidence of hip and other osteoporotic fractures. Osteoporos Int.

[7] Cummings SR, Melton LJ (2002). Epidemiology and outcomes of osteoporotic fractures. Lancet.

[8] Haidukewych GJ, Berry DJ (2004). Nonunion of fractures of the subtrochanteric region of the femur. Clin Orthop Relat Res.

[9] Haidukewych GJ, Israel TA, Berry DJ (2001). Reverse obliquity fractures of the intertrochanteric region of the femur. J Bone Joint Surg.

[10] Hoskins W, Bingham R, Joseph S, Liew D, Love D, Bucknill A, Oppy A, Griffin X (2015). Subtrochanteric fracture: the effect of cerclage wire on fracture reduction and outcome. Int J Care Inj.

[11] Hozo SP, Djulbegovic B, Hozo I (2005). Estimating the mean and variance from the median, range, and the size of a sample. BMC Med Res Methodol.

[12] Johnell O, Kanis JA (2006). An estimate of the worldwide prevalence and disability associated with osteoporotic fractures. Osteoporos Int.

[13] Kannus P, Parkkari J, Sievänen H, Heinonen A, Vuori I, Järvinen M (1996). Epidemiology of hip fractures. Bone.

[14] Karayiannis P, James A (2020). The impact of cerclage cabling on unstable intertrochanteric and subtrochanteric femoral fractures: a retrospective review of 465 patients. Eur J Trauma Emerg Surg.

[15] Kasha S, Yalamanchili RK (2020). Management of subtrochanteric fractures by nail osteosynthesis: a review of tips and tricks. Int Orthop (SICOT).

[16] Kennedy MT, Mitra A, Hierlihy TG, Harty JA, Reidy D, Dolan M (2011). Subtrochanteric hip fractures treated with cerclage cables and long cephalomedullary nails: a review of 17 consecutive cases over 2 years. Int J Care Inj.

[17] Kilinc BE, Oc Y, Kara A, Erturer RE (2018). The effect of the cerclage wire in the treatment of subtrochanteric femur fracture with the long proximal femoral nail: a review of 52 cases. Int J Surg.

[18] Meinberg EG, Agel J, Roberts CS, Karam MD, Kellam JF (2018). Fracture and dislocation classification compendium-2018. J Orthop Trauma.

[19] Moher D, Liberati A, Tetzlaff J, Altman DG, Group P (2009). Preferred reporting items for systematic reviews and meta-analyses: the PRISMA statement. J Clin Epidemiol.

[20] Nieves JW, Bilezikian JP, Lane JM, Einhorn TA, Wang Y, Steinbuch M (2010). Fragility fractures of the hip and femur: incidence and patient characteristics. Osteoporos Int.

[21] Papadimitriou N, Tsilidis KK, Orfanos P, Benetou V, Ntzani EE, Soerjomataram I (2017). Burden of hip fracture using disability-adjusted life-years: a pooled analysis of prospective cohorts in the CHANCES consortium. Lancet Public Health.

[22] Patil R, Modi S, Rajoli S, Kumar R, Ghelani G (2019). Effect of encerclage wiring with intermedullary nailing in subtrochanteric fractures of femur. Indian J Orthop Surg.

[23] Rehme J, Woltmann A, Brand A (2021). Does auxiliary cerclage wiring provide intrinsic stability in cephalomedullary nailing of trochanteric and subtrochanteric fractures?. Int Orthop (SICOT).

[24] Stang A (2010). Critical evaluation of the newcastle-ottawa scale for the assessment of the quality of nonrandomized studies in meta-analyses. Eur J Epidemiol.

[25] Starr AJ, Hay MT, Reinert CM, Borer DS, Christensen KC (2006). Cephalomedullary nails in the treatment of high-energy proximal femur fractures in young patients: a prospective, randomized comparison of trochanteric versus piriformis fossa entry portal. J Orthop Trauma.

[26] Trikha V, Das S, Agrawal P, Arkesh M, Dhaka SK (2018). Role of percutaneous cerclage wire in the management of subtrochanteric fractures treated with intramedullary nails. Chin J Traumatol.

[27] Wähnert D, Lenz M, Schlegel U, Perren S, Windolf M (2011). Cerclage handling for improved fracture treatment. A biomechanical study on the twisting procedure. Acta Chir Orthop ET Traumatol Cechoslov.

