# Supplementary material for: The role of cerclage wiring in the management of subtrochanteric and reverse oblique intertrochanteric fractures: a meta-analysis of comparative studies
Source: Eur J Orthop Surg Traumatol. 2022 Mar 21;33(4):739–49. doi: 10.1007/s00590-022-03240-z (PMC10125946; doi:10.1007/s00590-022-03240-z)
Supplement: Supplementary file 1 — Supplementary file1 (DOCX 113 KB) [file 590_2022_3240_MOESM1_ESM.docx]

**Supplementary**

Supplementary Table 1: Qualitative Analysis of Included Studies.

| Study | Selection | Comparability | Outcome |
| --- | --- | --- | --- |
| Annappa, 2020 [1] | *** | * | *** |
| Bhat, 2019 [4] | *** | * | ** |
| Patil, 2019 [22] | *** | ** | ** |
| Trikha, 2018 [26] | *** | ** | *** |
| Codesido, 2017 [5] | *** | ** | *** |
| Hoskins, 2015 [10] | *** | * | * |

Supplementary Figure 1: Pooled prevelance of Non-delayed union in IMN-C group.

The dashed line represent the line of overall effect.

Supplementary Figure 2: Pooled prevelance of Non-delayed union in IMN group.

The dashed line represent the line of overall effect.

Supplementary Figure 3: Pooled prevelance of good reduction in IMN-C group.

The dashed line represent the line of overall effect.

Supplementary Figure 4: Pooled prevelance of good reduction in IMN group.

The dashed line represent the line of overall effect.

Supplementary Figure 5: Pooled prevelance of Acceptable reduction in IMN-C group.

The dashed line represent the line of overall effect.

Supplementary Figure 6: Pooled prevelance of Acceptable reduction in IMN group.

The dashed line represent the line of overall effect.

Supplementary Figure 7: Pooled prevelance of Poor reduction in IMN-C group.

The dashed line represent the line of overall effect.

Supplementary Figure 8: Pooled prevelance of Poor reduction in IMN group.

The dashed line represent the line of overall effect.

Supplementary Figure 9: Pooled prevelance of Superficial Infection in IMN-C group.

The dashed line represent the line of overall effect.

Supplementary Figure 10: Pooled prevelance of Superficial Infection in IMN group.

The dashed line represent the line of overall effect.

Supplementary Figure 11: Pooled prevelance of Deep Infection in IMN-C group.

The dashed line represent the line of overall effect.

Supplementary Figure 12: Pooled prevelance of Deep Infection in IMN group.

The dashed line represent the line of overall effect.

Supplementary Figure 13: Pooled prevelance of Leg length discrepency in IMN-C group.

The dashed line represent the line of overall effect.

Supplementary Figure 14: Pooled prevelance of Leg length discrepency in IMN group.

The dashed line represent the line of overall effect.

Supplementary Figure 15: Pooled prevelance of Implant Failure in IMN-C group.

The dashed line represent the line of overall effect.

Supplementary Figure 16: Pooled prevelance of Implant Failure in IMN group.

The dashed line represent the line of overall effect.

Supplementary Figure 17: Pooled prevelance of Screw cut out in IMN-C group.

The dashed line represent the line of overall effect.

Supplementary Figure 18: Pooled prevelance of Screw backout in IMN-C group.

The dashed line represent the line of overall effect.

Supplementary Figure 19: Pooled prevelance of Screw cut out in IMN group.

The dashed line represent the line of overall effect.

Supplementary Figure 20: Pooled prevelance of Screw cut out in IMN group.

The dashed line represent the line of overall effect.

Supplementary Figure 21: Pooled prevelance of Reoperation and Revision in IMN-C group.

The dashed line represent the line of overall effect.

Supplementary Figure 22: Pooled prevelance of Reoperation and Revision in IMN group.

The dashed line represent the line of overall effect.
